# Research on student engagement in distance learning in sustainability science to design an online intelligent assessment system

**DOI:** 10.3389/fpsyg.2023.1282386

**Published:** 2023-11-23

**Authors:** Kailun Fang, Li Li, Yifei Wu

**Affiliations:** ^1^Guangzhou Urban Planning and Design Co., Ltd., Guangzhou, China; ^2^Shanghai Baiaoheng New Material Co., Ltd., Shanghai, China; ^3^School of Architectural Engineering, Shenzhen Polytechnic University, Shenzhen, China

**Keywords:** student engagement, engagement assessment system, distance learning, higher education, machine learning algorithms

## Abstract

Distance learning programs in sustainability science provide a structured curriculum that covers various aspects of sustainability. Despite the growing recognition of distance learning in higher education, existing literature has primarily focused on specific and detailed factors, without a comprehensive summary of the global themes, especially neglecting in-depth exploration of poor engagement factors. This study bridged this gap by not only examining detailed factors but also synthesizing the overarching themes that influenced student engagement. The aim of this study was to investigate the factors that impact student engagement in distance learning within higher education institutions across different countries. By developing a theoretical framework, three key aspects of student engagement in higher education were identified. A total of 42 students and 2 educators affiliated with universities participated in semi-structured interviews. The findings of this paper indicated that sociocultural, infrastructure, and digital equity factors were the main influencing factors of student engagement. Furthermore, a student engagement assessment system was developed using machine learning algorithms to identify students with low levels of engagement and conduct further analysis that considers the three aforementioned factors. The proposed automated approach holds the potential to enhance and revolutionize digital learning methodologies.

## 1 Introduction

Sustainability science education is a dynamic and interdisciplinary field that equips students with the knowledge, skills, and mindset to address the complex challenges facing our planet today and in the future. Rooted in the principles of sustainability, this educational approach goes beyond traditional silos to bridge gaps between various scientific disciplines, social factors, and environmental considerations (Muhammad et al., 2022; Rigler et al., 2022; Saleh, 2022). At its core, sustainability science education seeks to empower individuals to become informed and responsible global citizens who can actively contribute to the well-being of both people and the planet. Distance learning (DL), also known as online education or e-learning, has revolutionized the way education is delivered and accessed. When applied to the field of sustainability science, distance learning offers a flexible and accessible avenue for individuals to engage with the principles, concepts, and practices of sustainability without the constraints of physical classroom attendance (Avsec et al., 2022). This mode of education opens up opportunities for a diverse range of learners, regardless of geographical location or scheduling limitations, to contribute to the global efforts toward a more sustainable future. Based on online learning and instruction, DL breaks space limits with a completely different philosophical and conceptual model of learning and allows students from various geographical areas to engage with an academic institution and other students online to pursue a degree or certification (Abuhammad, 2020). With the help of DL, students have the freedom to learn at their own pace and time. In terms of course design, assessment, and teaching strategies, DL is different from online learning before the unprecedented pandemic, in which the latter was originally designed for face-to-face teaching (Tsarapkina et al., 2020). An increasing number of institutions and educators have decided to use developing technology to provide educational services and engage with students via online platforms due to the advantage of DL (Stenman and Pettersson, 2020).

Although academic institutions and educators have acknowledged the importance of DL, many challenges have been observed in various countries. The most widely discussed challenge by researchers is student engagement, which refers to a student’s connection with the learning environment (Al-Balas et al., 2020). Many researchers have made great efforts to explore the influencing factors for student engagement from different aspects. Some studies have focused on students’ behaviors from the perspective of physical engagement based on physiological and behavioral data output by new biometric tools. With abundant experimental analyses using hand sensors (Morrison et al., 2020), wearable sensors (Carroll et al., 2020), heart rate sensors (Senthil and Wong, 2017), eye-tracking (Wang et al., 2021) etc., these research have developed and tested a series of measurement and classification technique to assess students engagement. Another group of researchers placed the emphasis on students’ online learning experiences. They maintained in distance education, student participation plays a critical role in learning and personal development, as it is recognized as one of the best indicators of learning progress. The more students engage with a subject in sustainability science education, the more they can learn (Kaplan and Haenlein, 2016). However, Several studies have shown that students’ learning experience in DL is influenced by various factors, including the detached and non-interactive nature of lectures, lack of concentration, and more (Arinto, 2016; Liu et al., 2019; Lapada et al., 2020). Regardless of their circumstances, distance learners are susceptible to missing social interaction and showing little participation, leading to unsatisfactory learning experiences, like feelings of isolation, disconnectedness and helplessness (Demouy et al., 2016; Kusmaryono et al., 2021).

Although many studies have identified the underlying factors of student engagement in DL from their different analytical angles and perspectives, they have primarily focused on specific and detailed factors. These existing studies lack a comprehensive and systematical exploration of the underlying factors related to student engagement, especially in-depth exploration of poor engagement factors (Gan et al., 2007; Huang et al., 2012; Demouy et al., 2016; Lee et al., 2019). Furthermore, while some studies have proposed strategies for DL in primary education (Vanderlinde et al., 2010; Edisherashvili et al., 2022), they have paid little attention to higher education and the utilization of algorithm systems for automated assessment. Even for some researchers applying machine learning to investigate student engagement, they only focuses on single-dimension index, like activity logs, student attendance, behavioral data, etc. (Orji and Vassileva, 2020). These gaps are what prompted this study to explore the implementation of DL in higher education. Specifically, this research aims to achieve three goals: (1) To explore the factors that influence student engagement in DL from the perspectives of students; (2) To develop a model for student engagement in different course activities through the utilization of machine learning algorithms; (3) To promote sustainability science in DL.

The relevance of the findings are as follows: (1) Understanding effective DL strategies provides valuable insights for educational institutions to be better prepared for sustainability science education. (2) The experiences and findings from DL can inform the development of blended learning models that combine in person and remote instruction. Educational systems may adopt hybrid approaches that integrate elements of DL to enhance flexibility, personalized learning, and educational outcomes. (3) The DL on sustainability science education highlighted the importance of technology in education. The findings from DL can inform decisions regarding technology integration, infrastructure improvements, and digital literacy training to ensure that institutions are equipped to provide effective and inclusive education in the digital era.

The justification for Chinese DL on sustainability science education is that China’s vast geographical expanse and population density make traditional education challenging to deliver uniformly to all regions. DL allows for widespread access to quality sustainability education, enabling individuals from urban centers to rural communities to participate and contribute to sustainable practices. As China is a global leader in technology adoption. DL can leverage cutting-edge technologies like virtual reality, augmented reality, and artificial intelligence to create immersive and engaging learning experiences that resonate with tech-savvy learners (Asif et al., 2020; Yu et al., 2020; Liu and Li, 2021). As China lacks DL courses on sustainability science education in higher education, many people seek to take online courses in overseas universities. Improving the education quality on sustainability science can better promote the sustainable development goals in China and raise awareness of sustainable concepts within the country (Rudenko et al., 2020).

This paper is organized into six sections. The next section proposes a theoretical framework based on relevant theoretical aspects obtained from previous work. This is followed by section 3, which outlines the methodology of the study. In section 4, the research findings of the study are presented. Section 5 focuses on the development of a student engagement assessment system. Finally, Section 6 concludes the paper.

## 2 Literature review and theoretical framework

### 2.1 Literature review

This work builds on related research that grounded our research in the context of supporting DL and student engagement in DL. DL, refers to the delivery of educational instruction through digital devices with the purpose of facilitating learning, including the ability to study from any location at any time, the potential for substantial cost savings, the elimination of commuting on crowded buses or local trains, the flexibility to choose one’s learning schedule, and time-saving advantages (Al-Balas et al., 2020; Ferri et al., 2020). Social interaction and learning effectiveness in DL are important and it has faced many challenges because of technical, personal, logistical and financial barriers (Abuhammad, 2020). Engagement has been recognized as a significant precursor to academic success (Lee et al., 2019). Student engagement is a prominent term in higher education, now extensively studied, theorized, and discussed, with mounting evidence of its pivotal role in academic success and learning (Kahu, 2013). Student engagement comprises both the time students dedicate to reading online materials and their utilization of online features. Active engagement, on the other hand, involves students learning through experimentation and collaborative work, which is a key factor in student engagement in DL (Huang et al., 2012). Student engagement encompasses behavioral, psychological, and cognitive dimensions (Lee et al., 2019).

Previous studies on student engagement in DL have primarily focused on physical issues and online learning experiences, without systematically exploring the factors behind the negative engagement. This gap limits researchers from obtaining a complete and logical understanding of DL, including its antecedents and consequences (Caballé et al., 2010; Huang et al., 2012; Demouy et al., 2016). Furthermore, while some studies have proposed strategies for DL in primary education (Vanderlinde et al., 2010; Edisherashvili et al., 2022), they have paid little attention to higher education and the utilization of algorithm systems for automated assessment. These gaps are what prompted this study to explore the implementation of DL in higher education, and existing research has little solution for the low student engagement of DL. Important differences between our efforts and these past precedents include a different in methodological approach. Moreover, this study proposed a solution by utilization of algorithm systems for automated assessment in the future.

### 2.2 Theoretical framework

DL in sustainability science can serve as a valuable resource for professionals seeking to enhance their knowledge and skills in sustainable practices. It offers opportunities for career advancement and diversification. Interestingly, DL aligns with sustainability goals by reducing the need for commuting and physical resources associated with traditional classrooms, thus minimizing the carbon footprint of education. While DL of sustainability science offers numerous benefits, it is important to acknowledge potential challenges such as digital accessibility, self-discipline, and the need for robust online infrastructure. Institutions and educators must strive to create inclusive and engaging online environments that foster deep understanding and meaningful contributions to sustainability efforts worldwide. And in the DL of sustainability education, the student engagement is important to promote the sustainability science and sustainable goals.

In this study, student engagement is defined as encompassing cognitive, behavioral, and emotional components, and it is best understood as a relationship between students and their surroundings, which includes the community and individuals within the institution, the instruction they receive, and the curriculum they engage with (Almajali et al., 2022). It has become a prominent term in higher education, leading to increased research, theorization, and debates, owing to mounting evidence of its significant role in achievement and learning (Appolloni et al., 2021). In general, student involvement is considered one of the strongest predictors of learning and personal development, as the more students actively participate in a subject, the more they are likely to learn (Dixson, 2015). Similarly, the more students receive feedback on their learning, the more proficient they can become (Zhoc et al., 2018).

Student engagement is complex and multifaceted. There is debate over the exact nature of the construct, and a key problem is the lack of distinction between the state of engagement, its antecedents, and its consequences in DL. While there is some overlap, three relatively distinct approaches to understanding engagement can be identified in the literatures: (1) The socio-cultural perspective considers the critical role of the socio-cultural context. This perspective has proven valuable for examining both the learning processes of individual students and broader educational changes within socially situated studies of development and educational transformation. It can be employed to explore how DL is influenced by the values and norms within a community of learners. This perspective is valuable for DL as it recognizes that learners are social beings, and their development is shaped by interactions within their learning environment. Consequently, the learning activities used in DL may also be beneficially approached from the socio-cultural perspective. (2) Infrastructure is a valid and reliable measure to assess e-learning system success and is a foundation for achieving the success of DL systems. (3) Digital equity, inclusive education, culturally responsive teaching, new technologies, virtual world learning communities, and intersectionality are concepts that can either facilitate or impede the connection of individuals to new learning experiences and knowledge-building opportunities. This interconnectedness poses a challenge to researchers, teachers, educators, school leaders, policymakers, and institutions as they reassess their existing work (Table 1).

**Table 1:** Factors influencing the student engagement in the literature.

Factors	References
Socio-cultural	Davis and Niederhauser (2005)
Infrastructure	Kahu (2013) and Moore and Fodrey (2017)
Digital equity	Aguilar (2020) and Pittman et al. (2021)

**Figure 1:**
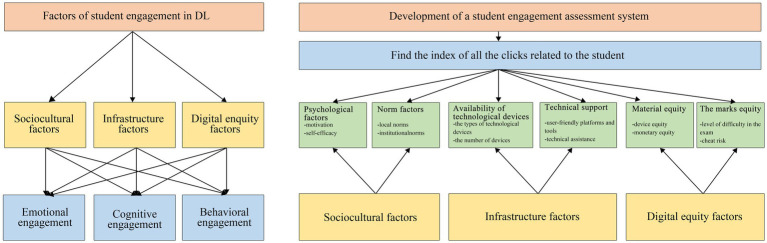
Theoretical framework (drawn by authors).

By summarizing the global themes that influence student engagement, factors in DL refer to the various elements and influences that affect students’ active involvement, motivation, and interaction in the online learning environment. These factors can significantly impact students’ learning outcomes and overall educational experience. In DL, student engagement factors relate to cognitive (mental involvement), behavioral (active participation), and emotional (affective connection) engagement. Cognitive engagement is related to factors including active learning strategies, meaningful content, and reflection. Behavioral engagement-related factors include interactivity, clear guidance, and time organization. Emotional engagement-related factors include instructor presence/support, peer interaction/social presence, and personal relevance. Consideration of these factors helps in creating an engaging DL environment that fosters students’ active involvement, motivation, and success (Figure 1). This framework aimed to enhance the understanding of the causes and consequences of DL in higher education, addressing the gaps in the existing research. The emphasis on identifying global themes can lead to a more holistic understanding of the factors that impact student engagement across different contexts and educational settings. This research contributes to the field by providing valuable insights that can inform educational policies and practices to enhance student engagement and overall academic outcomes.

## 3 Research methods

The purpose of this study was to investigate the factors affecting student engagement in DL in higher education. The following research questions guided the study: (1) What are the factors that influence student engagement in the DL? (2) Can student engagement in different course activities be modeled by utilizing machine learning algorithms?

Qualitative data were collected through semi-structured interviews to understand the DL and students’ engagement, allowing situational analysis across the context and enabling scholars to identify similarities between the cases. The characteristics of the semi-structured interview are as follows: (1) There are certain themes, and the structure of questions is loose, but there is still some focus and focus, not rambling; (2) Prepare interview outline or interview points before the interview but ask questions. New questions can be formed at any time during the interview according to the interviewee’s answers. When put forward, there is considerable elasticity; (3) The interviewer does not need to use specific words or semantics for the interview. Based on the theoretical framework, deductive content analysis was performed with similar ideas grouped together to form main categories and generic categories for making valid inferences from data to their context. As a result, three aspects were effectively identified and confirmed as significant: the sociocultural perspective, the infrastructure perspective, and the digital equity perspective.

### 3.1 Data collection

In accordance with the study’s objectives, two out of the four proposed approaches were employed: both face-to-face and online methods were used for all 44 participants. The semi-structured interviews adhered to Patton’s general interview guidelines. As a result, the interview guide was divided into two main sections, each addressing the subjects of DL and participants’ feelings regarding the adoption of DL. Furthermore, we developed a list detailing the topics and questions to be covered as initially suggested. Potential interviewees were identified through personal connections or referrals. Following this, invitation letters were sent to them. Individuals recommended by other students were given the option to accept or decline the invitation to participate in the interview process.

Subsequently, the interview process commenced with all the participants. The open-ended portion of the interview focused on gathering background information about the interviewees or participants, their past experiences, and feedback related to DL to enhance energy performance. This step significantly contributes to the validity of the interview process by assessing whether the interviewees possessed the necessary experience to support the data. The second section concentrated on existing building renovations and the challenges associated with adopting DL.

For the face-to-face interviews, the process began with an introduction of the interviewer, followed by questions on various topics related to sustainable technology and existing building renovations. Commencing with open-ended questions allowed participants to freely express their thoughts, giving them a general idea of what the interview would entail. This approach also created a welcoming and friendly environment, which boosted participants’ confidence levels. However, it is worth noting that the order of topics in the guide was not strictly adhered to, as semi-structured interviews allow participants the freedom to contribute without strictly following a predefined script.

### 3.2 Procedure

Upon completion of the interview guide design, the interviewer selected 5–10 individuals from the target group for a pretest. Following the testers’ completion of the survey, the notes from each session were reviewed. Typically, this review phase revealed the primary issues, allowing the interviewer to enhance the survey to effectively tackle these identified problems. Individual interviews adopted the following questions: (1) Give the marks on the five-point (1–5) active engagement scale; (2) Does DL provide a good experience and participation for you on sustainability science education? (3) What influences students’ participation with educators? (4) Is it easier for the respondent to achieve high marks in DL? (5) What suggestions do you have for the future development of DL in sustainability science education? The questions were cross-referencing the questions chosen with validation from the literature reviewed (Table 2). 44 researchers conducted the interviews, and an interview protocol was developed. Individual meetings lasted about 30 min on average. The interviews were held in the interviewees’ native languages.

**Table 2:** Cross-referencing the questions chosen.

Question	Literature review
Give the marks on the five-point (1–5) active engagement scale.	Active engagement entails a high level of involvement and a profound understanding and expertise in various activities (Starr-Glass, 2020; Ladino Nocua et al., 2021).
(2) Does DL provide a good experience and participation for you on sustainability science education?	experience and participation were performed on some affective and interaction parameters derived from the answers of a semi-structured survey (Capone and Lepore, 2021).
(3) What influences students’ participation with educators?	Students’ levels of participation in DL, distinguishing between different analyses deploying varied theoretical frameworks, including sociology, social psychology studies in science major (Henriksen, 2015; Park, 2014).
(4) Is it easier for the respondent to achieve high marks in DL?	Studies indicated that students reported significantly higher scores in DL and DL may obtain better results (Arbaugh and Benbunan-Finch, 2006).
(5) What suggestions do you have for the future development of DL in sustainability science education?	Distance learning is an opportunity to enhance continuing professional development in sustainability science (Southernwood, 2008; Henriksen, 2015).

### 3.3 Participants

Participants in this study were drawn at random using a purposive and convenience sampling approach, with researchers relying on participant referrals to find new participants. The process was carried out with participants to ensure that they were aware of the nature of their participation and that it was voluntary and confidential. The sample population was university graduates from China who joined the DL, which is held by different countries, like Australia, Singapore, Malaysia, the USA, the UK, Hong Kong China, Mainland China, etc. (Table 3). Their majors have included Arts, Urban Planning, and Economy. There was a total of 44 students, comprising 24 females and 20 males. The students’ ages ranged from approximately 20 to 24 years old, with the majority being full-time students. Additionally, the educators’ ages were approximately in the range of 30 to 35 years old. Since it was unknown how many students have experienced DL, participants were recruited through convenience sampling with a number of 44.

**Table 3:** The information from interviewees.

Number	Course provided country	Interviewees	Nationality	Gender
1	Malaysia	S1	Chinese	Female
2	Malaysia	S2	Chinese	Male
3	Malaysia	S3	Chinese	Male
4	Malaysia	S4	Chinese	Male
5	Malaysia	S5	Chinese	Female
6	Hong Kong	S6	Chinese	Male
7	Hong Kong	S7	Chinese	Female
8	Hong Kong	S8	Chinese	Female
9	Hong Kong	S9	Chinese	Male
10	Hong Kong	S10	Chinese	Male
11	Australia	S11	Chinese	Male
12	Australia	S12	Chinese	Female
13	New Zealand	S13	Chinese	Female
14	Singapore	S14	Chinese	Male
15	Japan	S15	Chinese	Male
16	Japan	S16	Chinese	Male
17	Japan	S17	Chinese	Female
18	Japan	S18	Chinese	Female
19	New Zealand	E1	Chinese	Male
20	America	S19	Chinese	Female
21	America	S20	Chinese	Male
22	America	S21	Chinese	Male
23	America	S22	Chinese	Female
24	America	S23	Chinese	Female
25	America	S24	Chinese	Male
26	America	S25	Chinese	Male
27	UK	S26	Chinese	Female
28	UK	S27	Chinese	Female
29	UK	S28	Chinese	Male
30	UK	S29	Chinese	Female
31	UK	S30	Chinese	Female
32	China	S31	Chinese	Female
33	China	S32	Chinese	Male
34	China	S33	Chinese	Female
35	China	S34	Chinese	Female
36	China	S35	Chinese	Female
37	China	S36	Chinese	Female
38	China	S37	Chinese	Female
39	China	S38	Chinese	Male
40	China	S39	Chinese	Female
41	China	S40	Chinese	Male
42	China	S41	Chinese	Female
43	China	E2	Chinese	Female
44	Belarus	S42	Chinese	Female

### 3.4 Analysis

Deductive content analysis was performed, with similar ideas grouped together to form main categories and generic categories for making valid inferences from data to their context, with the goal of providing knowledge, new insights, a representation of facts, and a practical guide to action to achieve a condensed and broad description of the phenomenon. Each category is named with content-specific words. Subcategories with similar events and incidents are grouped together to form categories, and categories are grouped together to form main categories.

## 4 Results

### 4.1 Overview of student engagements in DL

This portion of the text analyzed the idea of actively involving students in higher education. It was crucial to recognize that even though students encountered challenges and uncertainties during the lockdown, the participants in the survey responded positively on a five-point scale (ranging from 1 to 5) measuring active engagement (mean = 3.51, standard deviation = 0.669). This response was probably influenced by the fact that the sample consisted of individuals who voluntarily participated in the survey. Moreover, the average five-point scale of cognitive, emotional, and behavioral engagements are 2.8, 3.5, and 3.4, respectively.

According to the students, one of the primary advantages of DL is its flexibility. Students can engage with course content at their own pace and according to their personal schedules, accommodating work, family, and other commitments. Just like in traditional education, DL of sustainability science education includes hands-on projects and assignments that encourage learners to apply their knowledge to real world situations. These projects might involve analyzing local sustainability challenges, proposing solutions, or conducting virtual fieldwork.

### 4.2 Sociocultural factors influencing student engagement in DL

Using inductive content analysis, this study identified three influential factors on student engagement in DL based on the data collected from participants. These key factors are sociocultural factors, infrastructure, and digital equity. The findings highlighted the significance of these factors in shaping the level of student engagement in DL contexts. This research provided valuable insights into the complex dynamics and DL elements that impacted students’ active participation and involvement in DL during emergencies.

Sociocultural factors emerged as influential social and cultural forces that significantly impacted students’ feelings, attitudes, values, thoughts, and beliefs during the interviews. These factors encompassed various aspects, including religious issues and societal norms, and played a vital role in shaping student engagement in the context of DL. The interview records shed light on the intricate interplay between sociocultural influences and student engagement, emphasizing the need to consider these factors in understanding and enhancing students’ active participation in DL environments (see Tables 4, 5).

**Table 4:** Main categories and generic categories.

Main categories	Generic categories	Explanation
Factors influencing students’ engagement	Sociocultural factors	Social and cultural forces
	Infrastructure factors	Use devices, support, Internet
	Digital equity factors	Access to information technology

**Table 5:** Thematic analysis of student engagement in ERL.

Code	Basic theme	Organizing theme	Global theme
Feelings of negativity	Motivation	Psychological factors	Sociocultural factors
Enjoy learning			
Studying plan	Self-efficacy		
Differences between places	Local norms	Norm factors	
Guideline	institutional norms		
Attendance marking			
Desktop computers, laptops, smartphones, and tablets	The types of technological devices	Availability of technological devices	Infrastructure factors
Shared devices	The number of devices		
Challenges of connectivity	User-friendly platforms and tools	Technical support	
Technological challenges			
Software			
Platform usability	Technical assistance		
Helpdesk			
No budget	Device equity	Material equity	Digital equity factors
Expensive	monetary equity		
difficult exam	level of difficulty in the exam	The marks equity	
Open book exam	Cheat risk		
Communicate in the exam through social media			

#### 4.2.1 Organizing theme 1: psychological factors

First and foremost, motivation emerged as a crucial psychological sub-factor that exerted a significant influence on student engagement in DL. Within the DL context, students encountered distinct challenges, including reduced social interaction and heightened distractions. Motivation, in turn, was found to be influenced by several key factors, such as intrinsic interest in the subject matter, the perceived relevance of the learning material, and the presence of external rewards or incentives. Students who demonstrated intrinsic motivation possessing a clear understanding of the benefits associated with their learning and felt supported were more likely to exhibit higher levels of engagement in DL activities. These findings underscore the importance of fostering motivation in DL environments to enhance student engagement and maximize learning outcomes:

*I felt so lonely in the DL process because we seldom talk to each other at the beginning. I do not know the others even after the introduction part at the beginning because many classmates did not open the camera to me (S5, Malaysia).*

Secondly, self-efficacy played a significant role in student engagement within the context of DL. Self-efficacy refers to an individual’s belief in their own capability to succeed in each task or situation. In DL, students often faced challenges related to time management, resource access, and navigating online platforms. Students who exhibited higher levels of self-efficacy were more likely to actively engage in DL, as they possessed a strong belief in their ability to overcome obstacles and thrive in the online environment. Conversely, students with lower self-efficacy tended to feel overwhelmed or discouraged, leading to disengagement from the learning process. These findings highlighted the importance of fostering and bolstering students’ self-efficacy beliefs to enhance their engagement and success in DL settings.

*I had to solve many problems when anything happened online. It was a great challenge to me because no friends sat beside me. In one class, I tried to answer the educator, but I found the facility to be broken at that time, and I was so upset on that day (S13, New Zealand).*

#### 4.2.2 Organizing theme 2: norm factors

Firstly, local norms were identified as a sociocultural factor that had a negative impact on student engagement in DL. The variation in time zones across different countries posed challenges for students who had to adhere to the schedule of their destination for studying. As a result, these students found themselves studying late at night, leading to fatigue and reduced engagement. Additionally, some students faced limitations in their home environment, as they did not have enough empty rooms and excessive noise disrupted their learning experience. During the interviews, students expressed dissatisfaction with evening class times and preferred morning classes. These findings highlight the significance of considering local norms and accommodating students’ preferred study times to enhance their engagement and learning experience in DL.

*As I was not employed during my course attendance, I found that opting for morning classes were more convenient for me (S11, Australia).*

Secondly, institutional norms were identified as influential factors in student engagement within the context of DL. These norms were established by educational institutions and played a significant role in shaping students’ level of engagement. They encompassed guidelines pertaining to attendance, participation requirements, and the availability of academic support services. Institutions that fostered a culture of engagement, provided adequate resources for DL, and prioritized student well-being were more likely to cultivate higher levels of student engagement. These findings emphasize the importance of institutional policies and support systems in creating an environment conducive to student engagement in DL.

*As my university had strict guidelines for sustainability science education and attendance, it was not easy to take sick leave because I wanted to graduate. For example, we had to turn on the camera and answer the questions frequently; it was very tough for a nonnative speaker (S23, America).*

### 4.3 Infrastructure factors influencing student engagement in DL

Infrastructure factors encompassed the availability of technological devices, technical support, internet access, and other related elements. The quality and usability of technology and tools employed in DL play a crucial role in influencing cognitive engagement. User-friendly platforms, reliable internet connectivity, access to suitable software and resources, and effective communication tools are vital components that enable students to actively engage in the learning process. These infrastructure factors directly impact students’ ability to navigate and effectively utilize DL tools, thereby influencing their cognitive engagement and overall learning experience (see Tables 2, 3).

#### 4.3.1 Organizing theme 1: the availability of technological devices

Firstly, technological devices such as desktop computers, laptops, smartphones, and tablets played a crucial role in DL. However, during the initial phase of DL, there was a shortage of devices, which posed a significant challenge. Participants in the study mentioned that although they had devices, they were not sufficient to meet the needs of their entire family. This scarcity was particularly evident due to the sudden increase in remote work arrangements in early 2020, where multiple family members had to share a single laptop or device. As a result, there was a temporary shortage of digital devices, which had a negative impact on students’ ability to actively engage in DL.

*I thought the Apple pencil was very useful in the DL. The pencil charges using a male lightning connector. And only IOS devices have the female lightning port. But I knew that not everyone could buy this (S15, Japan).*

#### 4.3.2 Organizing theme 2: technical support

Firstly, user-friendly platforms and tools were identified as crucial factors in DL. Platforms that were intuitive, user-friendly, and provided clear instructions played a significant role in enhancing students’ ease of use and minimizing frustration. Furthermore, ensuring compatibility across various devices and operating systems promoted inclusivity and enabled students to access materials and actively participate in DL, irrespective of their technological setup. These findings underscore the importance of selecting and implementing user-friendly DL platforms and tools to facilitate a seamless and accessible learning experience for students.

*The DL software had a great attraction for me to learn. Some user-friendly platforms would enable me to learn happily because they reduced many mistakes, but I remember one time I could not find my recording button, resulting in me missing the course (S39, China).*

Secondly, technical assistance played a crucial role in DL. This encompassed the provision of a help desk or IT support system that students could rely on when encountering technical difficulties. Timely and responsive technical support was found to enhance students’ confidence in navigating online platforms and foster active engagement. However, the study revealed that many participants faced numerous technical challenges while attempting to access the online classroom. They reported a lack of support from the school’s help desk due to the quarantine measures imposed. Moreover, many students expressed frustration with submitting assignments or test results through online systems, as there was a lack of qualified personnel with the necessary technical expertise to assist them. These findings highlight the shared infrastructure-related challenges experienced by both students and educators in DL:

*Teachers failed to exchange students’ education and connection. The main reasons for this were that many students did not know how to use the right technology, did not have enough devices, did not take online learning seriously, and it was a practical problem for special needs. Special need requires physical learning and attention (S42, Belarus).*

### 4.4 Digital equity factors influencing student engagement in DL

To fully participate in society, democracy, and economy, all persons and communities must have access to information technology. This is known as digital equity. The DL deepened the digital inequality as reported by the participants in the study, consequently reducing student engagement. It seems sustainability science education had broadened the digital divide among students of all levels without preparation (see Tables 2, 3).

#### 4.4.1 Organizing theme 1: material equity

One sub-factor is device equity. Students did not have enough budget to buy extra digital cameras, microphones, and earphones or unable to upload due to the speed of the internet. When the DL was implemented, the digital devices used in normal classes were not enough for the DL:

*The educator told us to begin the DL. Here’s what you needed to make sure your podcast or stream sounds the best, from microphones to good headphones (S14, Singapore).*

Secondly, monetary equity. Monetary equity involves addressing the financial barriers that students may face in accessing DL resources. This includes considerations such as the cost of internet connectivity, software or application subscriptions, and digital learning materials. Financial constraints may hinder students’ ability to access essential resources and tools for DL, leading to reduced engagement. By addressing monetary equity, educational institutions can ensure that all students have the necessary resources to actively participate in DL.

*It was not cheap to get the Internet for me as I was in the rural district. The lack of universal and affordable access to the Internet may widen income inequality (S38, China).*

#### 4.4.2 Organizing theme 2: the marks equity

Firstly, the level of difficulty in the exam. From some people’s point of view, online exams seem to be easy to take because some software can help to find references effectively on the internet. Even though access to the open book seems flexible, critical thinking views are still the main part of considering the marks when taking exams online. By contrast, some interviewees think online exams are more difficult than paper ones. Online exams often feel harder because it allows very little flexibility in the technical environment; students cannot make notes on the question paper nor flip through the pages, and it is difficult to view multiple windows at the same time unless they have a very large device screen. Overall, online exams in actuality are not harder, but the environment certainly gives the perception that they are.

*It was also important to note that online exams saved lots of time, they were green (no printing and ink, no papers, paper disposal, shipping, etc.), and there was no need to book physical testing places and hire invigilators (S26, UK).*

Secondly, cheat risk. While students were indeed concerned about the increased potential for cheating associated with remote exams, some instructors’ attempts to curb cheating put students at a disadvantage, especially those who were genuinely trying to engage appropriately. But one of the interviewees did agree with this, she thought the marks were the same as physical classes:

*It may be more difficult to get a higher mark. My mark is low and I think it is not so efficient for me to learn in RL (S1, Malaysia).*

*Offline exams are way better than online ones. It prevents cheating and copying which most students resort to in an online exam. Moreover, it provides equal opportunity for each student to showcase their ability and knowledge (S40, China).*

## 5 Discussion

### 5.1 Policy implication

#### 5.1.1 Sociocultural strategies

It should be focused on tackling the psychological negative impact like loneliness. In this study, it was observed that students in DL expect better learning surroundings and more communication with educators, classmates, and students on campus, as the DL reduces the student’s engagement and further leads to feelings of loneliness and boredom. In light of this, the researchers propose that extra attention should be paid to ensure the negativity does not burden international students. To facilitate stronger bonding among the students in DL, the following concrete approaches could be considered: (i) Reopen closed public spaces or some places that can be open online with VR technologies; (ii) Provide guidelines and resource support for face-to-face gatherings in accordance with safety restrictions; (iii) Arrange different online social online events.

#### 5.1.2 Infrastructure strategies

Innovative teaching practices, such as flipped course design, instructional media libraries, and project-based assessments, developed to augment instruction during DL hold promise for long term improvements to teaching and learning, though they are not yet universally embraced. Innovative educators are encouraged to use technology like discussion forums and interactive notebooks to communicate with students. Technical support can also be conducted in a remote way to help educators or students to solve device problems. The recording of DL is also important for students to look back on if they miss the schedule.

#### 5.1.3 Digital equity strategies

In addition to the decline of interaction between students and educators in DL, the unprecedented pandemic is also negatively related to digital equity. For those that do not have enough money to prepare for DL, funding for buying devices should be allocated. Additionally, those who have no need for old or unused technology should be encouraged to donate it. Enlisting the support of Best Buy or some giant chain to the cause is also a probable effective strategy.

### 5.2 Development of a student engagement assessment system

Based on the detailed factors and global themes identified in the literature review, there arises a need to develop an advanced AI-based student engagement assessment system. This system would leverage the comprehensive understanding of the factors influencing student engagement to create a more sophisticated and accurate evaluation mechanism. The AI system will be designed to analyze various aspects of student engagement, taking into account both explicit and implicit indicators. By integrating machine learning algorithms and natural language processing techniques, the system can process and interpret various aspects of student engagement, including data of student performance, behavior patterns, and feedback, among others.

**Figure 2:**
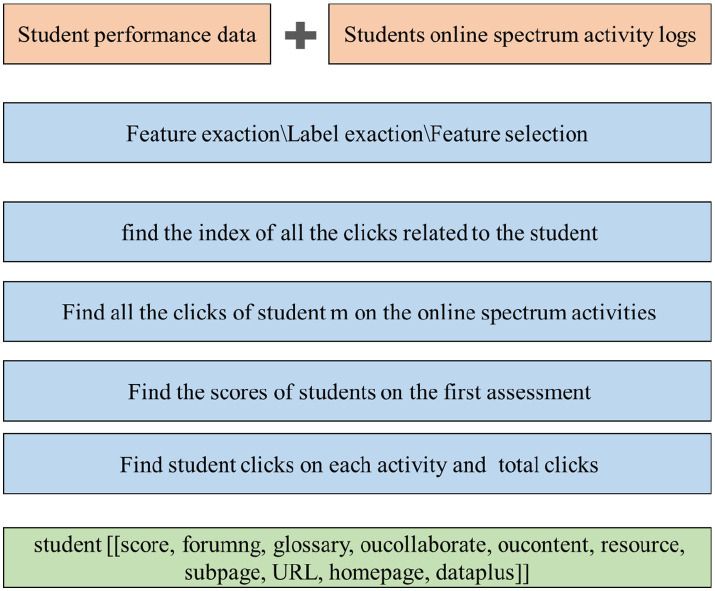
Algorithm framework of student engagement assessment system (drawn by authors).

**Figure 3:**
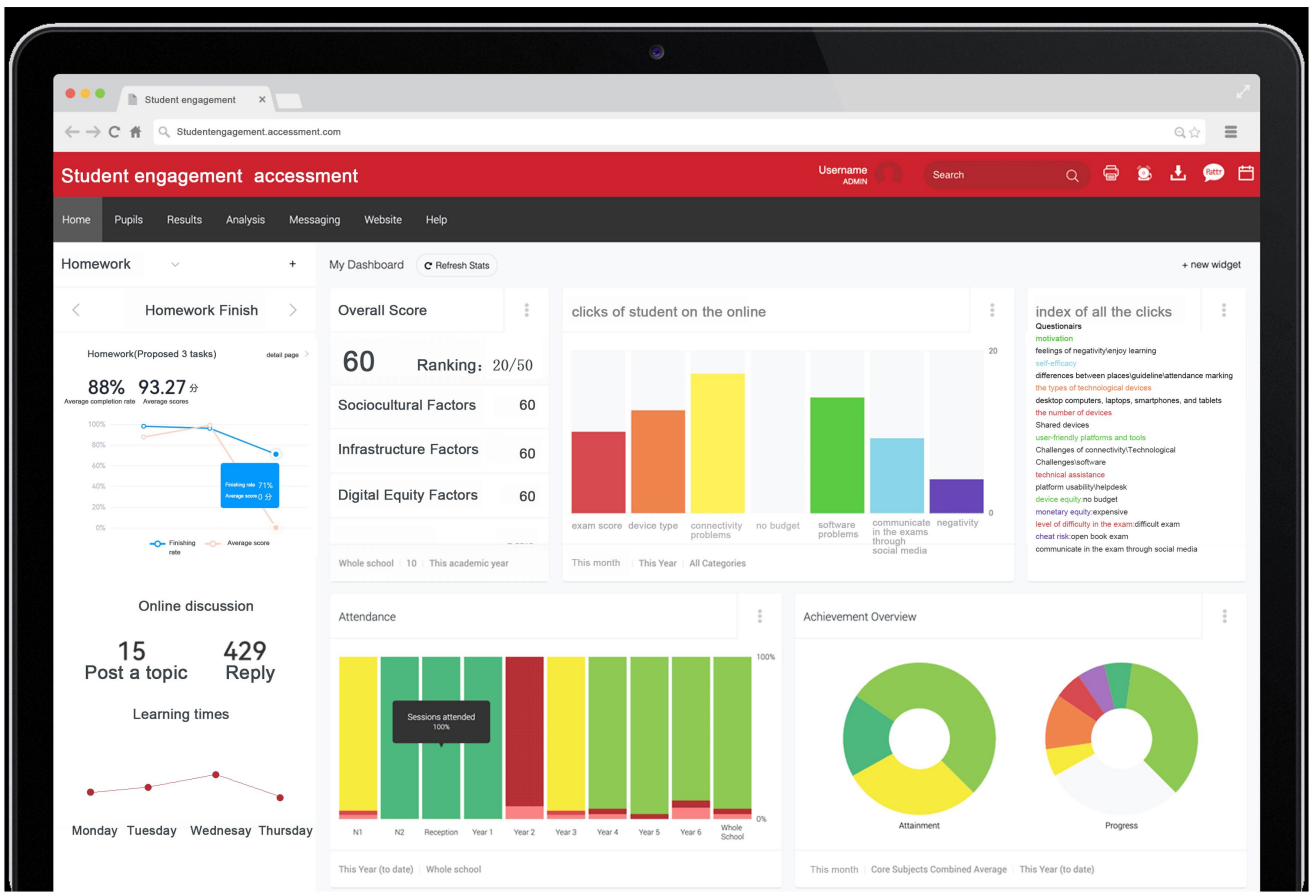
Diagrammatic drawing of engagement assessment system (drawn by authors).

An engagement prediction system has been designed to be inserted into the current students’ online spectrum. The main components of the proposed student engagement prediction system are detailed as follows: firstly, the system is a web-based system that offers students a variety of functions such as enrolling in courses, solving problems, completing assessments, downloading materials, and performing sustainability science education activities. The students can interact with the system daily to complete the sustainability science education course assessments for the classes in which they are enrolled. Secondly, when students interact with the system to complete a course assessment, their activities are recorded in the log file, and the student performance data are recorded in the student database. Thirdly, the preprocessing module extracts input-related features and engagement labels from the student log data and transform those data into a format acceptable for input into algorithms. Fourthly, based on the DL performances for the student log data, this module uses a machine learning algorithms model for making student engagement predictions to find low-engagement students in courses. Fifthly, the assessment is given to the students to find out which factors influenced their engagement. Lastly, a computer program that interprets these rules and displays them in the form of a graph provides valuable information about student engagement in DL activities to the instructor. The educators would be able to give advice to low-engagement students according to this predicted system (Figures 2, 3).

## 6 Conclusion and limitations

The importance of DL has been acknowledged among educators and researchers in various countries, with student engagement being widely discussed. However, previous studies primarily focused on specific and detailed factors in sustainability science education DL, such as physical engagement and online learning experiences, without systematically addressing the factors influencing student engagement, especially in-depth exploration of poor engagement factors. This research gap has limited researchers’ ability to obtain a comprehensive understanding of sustainability science education in DL. Furthermore, while existing studies on student engagement have offered insights into primary education, they have paid less attention to higher education and have not provided specific actions derived from lessons learned in that context.

By proposing a theoretical framework with a comprehensive summary of the global themes, this study identified three dimensions of student engagement in higher education during DL. This framework aimed to enhance the understanding of the causes and consequences of DL in higher education, addressing the gaps in the existing research. Semi-structured interviews were conducted with 42 students and 2 educators from various nationalities, studying or working in universities across different countries such as Australia, Singapore, Malaysia, the USA, the UK, Hong Kong, China, and Belarus. The results revealed a positive measurement of active engagement. Specifically, three factors influencing student engagement were identified: sociocultural factors, infrastructure factors, and digital equity factors.

The main contributions of this study are twofold: firstly, it identified factors related to student engagement in higher education of sustainability science in DL, namely, sociocultural factors, infrastructure factors and digital equity factors. The emphasis on identifying global themes can lead to a more holistic understanding of the factors that impact student engagement across different contexts and educational settings. This research contributes to the field by providing valuable insights that can inform educational policies and practices to enhance student engagement and overall academic outcomes. Secondly, with a comprehensive understanding of the factors influencing student engagement, this research developed an engagement assessment system using machine learning algorithms, which would benefit to the solution of a more sophisticated and accurate evaluation mechanism in the practice.

Despite its contributions, this study had inherent limitations in terms of sample size and participant characteristics. Conducting research with a larger sample size would enhance its validity. Moreover, further development of the student engagement system, incorporation of more machine learning models, and successful implementation in the online spectrums of university students should be pursued.

## Data availability statement

The original contributions presented in the study are included in the article/supplementary material, further inquiries can be directed to the corresponding author.

## Author contributions

KF: Conceptualization, Data curation, Investigation, Methodology, Project administration, Supervision, Validation, Formal analysis, Resources, Visualization, Writing – original draft. LL: Formal analysis, Investigation, Project administration, Writing – original draft. YW: Formal analysis, Funding acquisition, Investigation, Software, Writing – original draft, Writing – review & editing.
